# Alterations in Mucin-Associated Gene Expression on the Ocular Surface in Active and Stable Stages of Atopic and Vernal Keratoconjunctivitis

**DOI:** 10.1155/2021/9914786

**Published:** 2021-05-31

**Authors:** Mariko Horinaka, Jun Shoji, Akiko Tomioka, Yukiko Tonozuka, Noriko Inada, Satoru Yamagami

**Affiliations:** Division of Ophthalmology, Department of Visual Sciences, Nihon University School of Medicine, 30-1 Oyaguchi-Kamicho, Itabashi-ku, Tokyo 173-8610, Japan

## Abstract

**Purpose:**

To evaluate the presence of ocular surface mucin in patients with atopic and vernal keratoconjunctivitis (AKC/VKC), we investigated the mRNA expression levels of SAM-pointed domain-containing ETS-like factor (*SPDEF*) and mucin-related genes on the ocular surface.

**Methods:**

Nineteen patients with AKC or VKC were divided into two groups based on the severity of the disease as determined by their clinical scores for AKC/VKC: the stable group and the active group. Impression cytology was performed in all patients using filter paper, and the expression levels of *SPDEF*, *MUC1*, *MUC4*, *MUC5AC*, *MUC16*, and eotaxin-2 mRNA were determined by real-time reverse-transcription polymerase chain reaction.

**Results:**

The results showed that the expression levels of *SPDEF* and *MUC5AC* mRNA in the active group were significantly decreased compared with those in the stable group. Furthermore, clinical scores were significantly negatively correlated with the expression levels of *SPDEF* mRNA and significantly positively correlated with the expression levels of eotaxin-2, which is a biomarker for eosinophilic inflammation on the ocular surface. Cluster analysis classified the patients with AKC/VKC into three clusters, and the stable group was divided into two clusters according to the condition of ocular surface mucin.

**Conclusions:**

Ocular surface mucin in patients with AKC/VKC is altered in accordance with the clinical severity of the disease.

## 1. Introduction

Allergic conjunctival diseases are allergic disorders characterized by eosinophilic inflammation on the ocular surface (consisting of the tear film, conjunctiva, and cornea) and by an immediate hypersensitivity reaction with or without allergen-specific IgE antibodies [1]. Allergic conjunctival diseases consist of allergic conjunctivitis, atopic keratoconjunctivitis (AKC), and vernal keratoconjunctivitis (VKC) [2]; particularly, allergic conjunctival diseases with severe proliferative conjunctival lesions, including giant papillae and gelatinous limbal infiltration, are recognized as refractory and chronic forms of allergic conjunctival diseases that involve severe eosinophilic inflammation and type-2 inflammation via type-2 helper T cell (Th2)-mediated and group 2 innate lymphoid cell-mediated responses [3–5]. Apart from conjunctival proliferative lesions, the severe and chronic forms of atopic and vernal keratoconjunctivitis (AKC/VKC) are also characterized by corneal epithelial damage similar to that clinically observed in dry eye disease [6]. Therefore, maintaining a good tear film is an important aspect of a successful treatment in patients with chronic forms of AKC/VKC.

The three laminar structures of the precorneal tear film are the lipid, aqueous, and mucin layers [7]. Based on clinical tear tests, it has been reported that various allergic inflammation-associated molecules, including eosinophil cationic protein, cytokines, and chemokines, are present in tears [3, 8–10], which may cause corneal epithelial damage. Another ocular surface component closely related to corneal epithelial damage is mucin. Mucins are high-molecular-weight glycoproteins that contain a central region formed by tandem repeats and O-type carbohydrate chains bound to core proteins [11]. On the mucin-coated ocular surface, MUC5AC (as a gel-forming mucin), MUC1, MUC4, and MUC16 (membrane-associated mucins), as well as MUC7 (derived from the lacrimal gland), are present [12]. However, alterations in the mucin expression pattern on the ocular surface, in the presence of allergic inflammation, are not completely understood.

In patients with bronchial asthma, interleukin-13 (IL-13), a Th2 cytokine, has been shown to induce goblet cell hyperplasia in the respiratory epithelium. The induction of goblet cell differentiation is thought to upregulate the SAM-pointed domain-containing ETS-like factor (SPDEF) in respiratory epithelial cells [13, 14]. SPDEF is a transcription factor involved in goblet cell differentiation, and SPDEF-knockout mice show a decreased number of goblet cells in intestinal, bronchial, and conjunctival tissues [15]. Therefore, SPDEF in the respiratory epithelium is thought to be induced by intratracheal allergen exposure and Th2 cytokine stimulation [13, 14]. Furthermore, in allergic conjunctival diseases, goblet cells and MUC5AC secreted from goblet cells are decreased on the ocular surface of patients with AKC and shield ulcers [16]. In an allergic conjunctival mouse model, *MUC5AC* mRNA expression in the conjunctiva was found to be reduced after antigen instillation [17]. Therefore, the levels of ocular surface mucin are thought to fluctuate depending on the stage and severity of allergic conjunctival diseases [18].

In this study, we investigated the mRNA expression levels of goblet cell- and mucin-related genes, including *SPDEF*, *MUC5AC*, *MUC1*, *MUC4*, and *MUC16*, on the ocular surface in patients with VKC/AKC by impression cytology (i.e., the clinical ocular surface test). To clarify the allergic inflammatory effect on expression of ocular surface mucin and reduce statistical bias, we compared the results of the ocular surface test between patients with AKC/VKC in the active stage and those in the stable stage as controls. We also evaluated whether mRNA expression levels in goblet cell-related and mucin-related genes could provide useful results in ocular allergy tests.

## 2. Materials and Methods

This cross-sectional study was approved by the Institutional Review Board of the Nihon University School of Medicine (approval number: RK-120511-11) and adhered to the tenets of the Declaration of Helsinki. Written informed consent was obtained from all subjects enrolled in this study. This study conforms to the reporting requirements of the STROBE guidelines.

### 2.1. Patients

In total, 19 patients diagnosed with AKC or VKC at the Department of Ophthalmology of Nihon University Itabashi Hospital (Tokyo, Japan) were included in this study. The diagnosis of AKC and VKC was based on the Japanese guidelines for allergic conjunctival diseases [2]. The exclusion criteria were as follows: (1) the presence of ocular surface disease, including dry eye disease, other than allergic conjunctival diseases; (2) a history of treatment with systemic antiallergic or anti-inflammatory drugs, such as corticosteroids and immunosuppressants; (3) the use of eye drops to treat dry eyes, such as rebamipide or diquafosol sodium; and (4) the use of contact lenses.

We divided the cohort into 10 patients with stable-stage AKC/VKC (stable group) and nine patients with active-stage AKC/VKC (active group), according to their clinical scores. Patients in the stable group had clinical scores of <100 points, and patients in the active group had clinical scores of ≥100 points.

### 2.2. Clinical Scores

The 5-5-5 exacerbation grading scale is a clinical scoring system designed to determine the severity of allergic conjunctival diseases [19]. The clinical score is determined by summing the severe clinical findings, which are given 100 points per item, moderate clinical findings, which are given 10 points per item, and mild clinical findings, which are given 1 point per item. Severe clinical findings include active giant papillae, gelatinous infiltrates in the limbus, exfoliative epithelial keratopathy, shield ulcer, and papillary proliferation at the lower palpebral conjunctiva; moderate findings include blepharitis, papillary proliferation with velvety appearance, Horner–Trantas spots, edema of the bulbar conjunctiva, and superficial punctate keratopathy; and mild findings include papillae at the upper palpebral conjunctiva, follicular lesion at the lower palpebral conjunctiva, hyperemia of the palpebral conjunctiva, hyperemia of the bulbar conjunctiva, and lacrimal effusion. The range of clinical scores on the 5-5-5 exacerbation grading scale is 0 to 555 points.

### 2.3. Impression Cytology

The targeted eye for impression cytology is the affected eye in unilateral cases or the more severely affected eye in bilateral cases of AKC and VKC. Impression cytology specimens were obtained from the upper tarsal conjunctiva.

Impression cytology using the filter paper method was performed according to our previous reports [20–22]. Since the purpose of this study was to investigate the alterations in ocular surface mucin caused by allergic inflammation, we used the same method of eotaxin-2 mRNA expression level measurement in the upper tarsal conjunctiva as an ocular surface allergy test and thus collected specimens only from the upper tarsal conjunctiva. Briefly, the 5 mm tip of Schirmer's test paper (Schirmer Tear Production Measuring Strips; Ayumi Pharmaceutical Co., Tokyo, Japan) was used for impression cytology using the filter paper method. Schirmer's test paper was applied to the upper tarsal conjunctiva without local anesthesia, pressed gently using a glass rod, and then removed and preserved in RNA Stabilization Reagent (RNAlater; Qiagen, Hilden, Germany) until total RNA extraction. Then, total RNA in the impression cytology specimen was prepared using the RNeasy® Mini Kit (Qiagen) according to the manufacturer's information. cDNA was prepared using the High-Capacity cDNA Reverse-Transcription Kit (Life Technologies Japan, Carlsbad, CA, USA) according to the manufacturer's protocol.

### 2.4. Real-Time Reverse-Transcription Polymerase Chain Reaction

To detect the expression levels of *SPDEF*, *MUC1*, *MUC4*, *MUC5AC*, *MUC16*, and eotaxin-2 mRNA, the real-time reverse-transcription polymerase chain reaction (real-time RT-PCR) method was used. Real-time RT-PCR was performed using the TaqMan gene expression assay (Life Technologies) and predesigned primers/probes, including Hs00159367_m1 (*MUC1*), Hs00366414_m1 (*MUC4*), Hs00873651_m1 (*MUC5AC*), Hs01065189_m1 (*MUC16*), Hs01026050_m1 (*SPDEF*), and Hs00171082_m1 (eotaxin-2) (Life Technologies), on a StepOnePlus™ system (Life Technologies). Target Ct values were normalized to those of *GAPDH* (Hs99999905_m1) from the same sample. The relative expression levels of each target gene were determined using the ∆∆CT method.

### 2.5. Statistical Analysis

All statistical evaluations were performed using the MAC Toukei-Kaiseki v.2 software (Esumi, Tokyo, Japan). Differences in baseline characteristics between the active and stable groups were evaluated using Fisher's exact test or the Mann–Whitney *U* test. Expression levels of *SPDEF*, *MUC1*, *MUC4*, *MUC5AC*, and *MUC16* mRNA were compared between the stable and active groups using the Mann–Whitney *U* test. The relationships between the expression levels of *SPDEF*, *MUC16*, and eotaxin-2 mRNA were evaluated using a partial correlation coefficient test. Statistical significance was set at *p* < 0.05. Cluster analysis based on the Euclidian distance and Ward linkage was used to identify the grouping of chronic allergic conjunctival diseases patients using similar characteristics in terms of clinical scores and expression levels of *SPDEF*, *MUC16*, and eotaxin-2 mRNA.

## 3. Results

The demographic and clinical characteristics of the 19 patients in the stable and active groups are shown in Table 1. The average clinical score of the active group was significantly higher than that of the stable group. Eye drops containing an immunosuppressant were administered to all patients, and no patients were treated with steroid-containing eye drops. None of the patients changed their medications within the 3 months before or after the impression cytology test; even in stable-stage patients, the continuation of medications with immunosuppressive eye drops was necessary to maintain remission.

### 3.1. Expression Levels of SPDEF, MUC5AC, MUC1, MUC4, and MUC16 mRNA on the Ocular Surface

The results of real-time RT-PCR are ratios calculated using the ΔΔCT method, with *GAPHD* as the endogenous control and the reference patient's measurement as 1.

**Figure 1:**
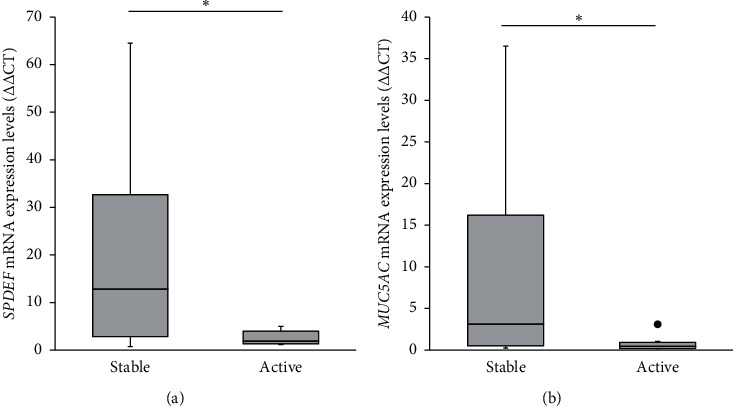
Messenger RNA expression levels of *SPDEF* and *MUC5AC* in stable and active groups. Expression levels of *SPDEF* (a) and *MUC5AC* (b) mRNA in the active group were significantly higher than those in the stable group. Data are presented as box-whisker plots. Significant differences are indicated as ^*∗*^*p* < 0.05.

**Figure 2:**
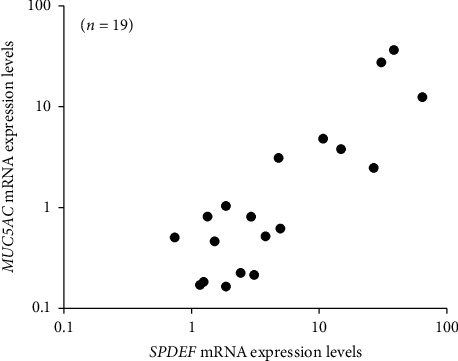
Correlation between *SPDEF* and *MUC5AC* mRNA expression levels. To examine the correlation, data from the active and stable groups were merged. Spearman's correlation coefficients showed significant correlations between *SPDEF* and *MUC5AC* mRNA expression levels (*ρ* = 0.770, *p*=0.000085).

**Figure 3:**
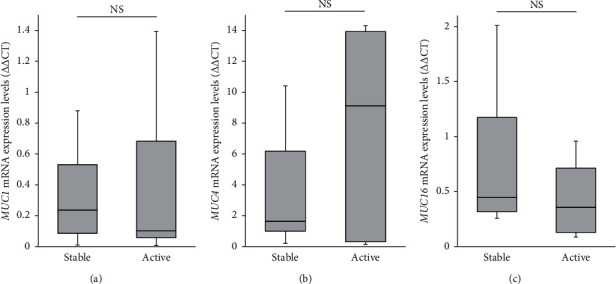
Messenger RNA expression levels of membrane-associated mucin in the stable and active groups. The expression levels of *MUC1* (a), *MUC4* (b), and *MUC16* (c) mRNA showed no significant (NS) differences between the active and stable groups.

The median values of *SPDEF* mRNA expression levels in the stable and active groups were 12.8 and 1.86, respectively. *SPDEF* mRNA expression levels in the active group were significantly lower than those in the stable group (*p* < 0.05, Mann–Whitney *U* test; Figure 1(a)). The median values of *MUC5AC* mRNA expression in the stable and active groups were 3.13 and 0.46, respectively. *MUC5AC* mRNA expression levels in the active group were significantly lower than those in the stable group (*p* < 0.05, Mann–Whitney *U* test; Figure 1(b)). *SPDEF* mRNA expression levels in the stable and active groups were significantly correlated with *MUC5AC* mRNA expression levels (*ρ* = 0.770, *p*=0.000085, Spearman's correlation coefficient; Figure 2). Significant differences were not observed between the stable and active groups in the expression of membrane-associated mucin mRNAs, including *MUC1*, *MUC4*, and *MUC16* (Figure 3).

In addition, eotaxin-2 mRNA expression levels in the stable and active groups were 30.7 (0.51–69.4) (median (range)) and 106 (3.63–433), respectively. Eotaxin-2 mRNA expression levels in the active group tended to be higher than those in the stable group; and the Mann–Whitney *U* test showed *p* < 0.05 for the one-tailed test and no significant difference in the two-tailed test between stable and active groups.

### 3.2. Relationship between Clinical Score and Goblet Cell- and Mucin-Related Gene Expression

**Figure 4:**
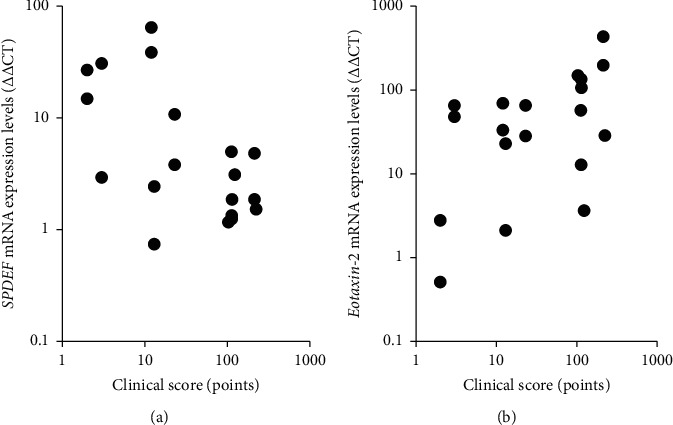
Correlations between clinical score and *SPDEF* mRNA expression and between clinical score and eotaxin-2 mRNA expression. Partial correlation coefficients showed significant correlations between clinical score and *SPDEF* mRNA expression levels (a) (*r* = −0.484, *p*=0.049) and between clinical score and eotaxin-2 mRNA expression levels (b) (*r* = 0.613, *p*=0.009).

The correlation between the clinical score and mRNA expression levels of *SPDEF*, *MUC16*, and eotaxin-2 was evaluated by determining the partial correlation coefficient. These values are presented in Table 2. The clinical score was significantly negatively correlated with the expression levels of *SPDEF* mRNA (*r* = −0.484, *p*=0.049; Figure 4(a)) and significantly positively correlated with the expression levels of eotaxin-2 mRNA (*r* = 0.613, *p*=0.009; Figure 4(b)).

With regard to *MUC5AC* mRNA expression levels, there was no correlation between eotaxin-2 and *MUC5AC* mRNA expression levels. In addition, there was a significant negative correlation between clinical score and *MUC5AC* mRNA expression levels (*ρ* = −0.536, Spearman's correlation coefficient).

### 3.3. Cluster Analysis of Patients with ACDs

**Figure 5:**
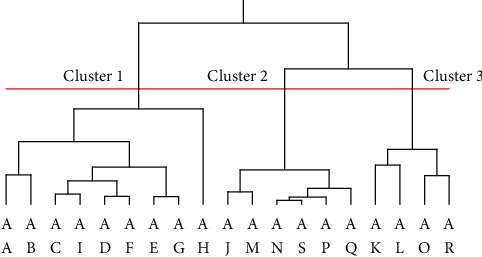
Dendrogram of cluster analysis in patients with chronic allergic conjunctival diseases (ACDs). Each of the 19 patients with chronic ACDs were given a code from AA to AS. The active group included those from AA to AI, whereas the stable ACD group included those from AJ to AS. The dendrogram depicts the levels of the hierarchical cluster.

All patients with active and stable AKC/VKC were evaluated by cluster analysis and grouped into three clusters according to their clinical score and expression levels of *SPDEF* and *MUC16* mRNA. The dendrogram is shown in Figure 5, and the characteristics of the three clusters are listed in Table 3. In the analysis of variance among the three clusters, significant differences were observed in the clinical score and expression levels of *SPDEF* and *MUC5AC* mRNA. Cluster 1 included all patients in the active group with low *SPDEF* and moderate *MUC16* mRNA expression. The stable group was divided into two subgroups: one with moderate *SPDEF* and low *MUC16* mRNA expression (Cluster 2) and one with high *SPDEF* and high *MUC16* mRNA expression (Cluster 3).

## 4. Discussion

We investigated the alterations in ocular surface mucin by performing a clinical ocular surface test using the impression cytology method in patients with AKC/VKC in the active stage. The results of the ocular surface test in the active group showed that the expression levels of *SPDEF* and *MUC5AC* mRNA were significantly decreased and that the mRNA expression levels of membrane-associated mucins, including *MUC1*, *MUC4*, and *MUC16*, were not significantly altered compared with those in the stable group. Furthermore, clinical scores showed a significant negative correlation with the expression levels of *SPDEF* mRNA and a positive correlation with the expression levels of eotaxin-2 mRNA. Cluster analysis clarified that patients with stable-stage AKC/VKC could be divided into two subgroups: a subgroup with moderate *SPDEF* and low *MUC16* mRNA expression and a subgroup with high *SPDEF* and high *MUC16* mRNA expression.

SPDEF is a transcriptional factor for goblet cell differentiation. It has been reported that SPDEF expression is increased at sites of goblet cell hyperplasia, which is caused by IL-13 and house-dust-mite allergens, in the respiratory epithelium of adult transgenic mice [12]. Our recent results, which were obtained by performing the clinical ocular surface test, showed that the expression levels of *SPDEF* mRNA were significantly decreased depending on AKC/VKC severity and were significantly correlated with the levels of *MUC5AC* mRNA. Secreted MUC5AC on the ocular surface originates in the conjunctival epithelium and is derived from goblet cells [16, 23]. In the conjunctiva of an experimental allergic conjunctivitis mouse model, the number of filled goblet cells reduced, and *MUC5AC* and *MUC4* mRNA levels decreased by the repeated instillation of allergens. Additionally, decreased goblet cell numbers returned to normal levels within 48 h after the last allergen instillation, and *MUC5AC* mRNA was found to be restored and increased 24 h after the last allergen instillation [18]. Regarding the expression of MUC5AC in allergic conjunctival diseases, Dogru et al. [16] reported that MUC5AC, secreted from goblet cells, is decreased in patients with AKC and shield ulcers. Based on our results and those of previous studies, severe allergic inflammation stimulated by allergen exposure on the ocular surface might decrease the number of goblet cells, and a reduction in MUC5AC secretion could elicit dry eye-like clinical findings. Furthermore, alterations in *SPDEF* and *MUC5AC* mRNA expression in the conjunctiva are thought to differ from those in the respiratory epithelium. Therefore, the expression levels of *SPDEF* mRNA, determined by the clinical ocular surface test, might be a useful tool to evaluate goblet cell density in the conjunctiva.

The clinical ocular surface test showed that the expression levels of membrane-associated mucins, including MUC1, MUC4, and MUC16, did not significantly differ between the active and stable groups. In our investigation on ocular surface mucin alterations in Sjögren's syndrome, we reported that *SPDEF* and *MUC16* mRNA expression levels are suitable markers for ocular surface mucin in the patients with Sjögren's syndrome [22]. Therefore, ocular surface mucin in this experiment was assessed with *SPDEF* and *MUC16* mRNA expression levels and dry eye. As a result, cluster analysis revealed that the stable group included a subgroup with moderate *SPDEF* and low *MUC16* mRNA expression levels and a subgroup with high *SPDEF* and high *MUC16* mRNA expression levels. In addition, the active group had low *SPDEF* and *MUC16* mRNA expression levels based on the clinical ocular surface test. These results indicate that chronic allergic conjunctival diseases can be divided into at least three clinical stages depending on the condition of the ocular surface mucin by simultaneously measuring *SPDEF* and *MUC16* through the ocular surface test. In a previous report, Dogru et al. [24] revealed that *MUC16* mRNA expression was significantly upregulated, whereas *MUC5AC* mRNA expression was significantly downregulated in the eyes of patients with AKC compared with that in the eyes of control subjects. This discrepancy in *MUC16* mRNA expression might be due to differences in the clinical stages of patients with AKC/VKC in each subjective group.

In this study, clinical scores were assessed based on objective findings using the 5-5-5 exacerbation grading scale for allergic conjunctivitis disease [19]. This scoring system is used to evaluate the severity of allergic conjunctival diseases. In the current study, the clinical scores of patients with AKC/VKC were positively correlated with eotaxin-2 mRNA expression [21]. Eotaxin-2 is a chemokine that is strongly expressed in eosinophils [20]. We have previously reported that expression levels of eotaxin-2 mRNA are a useful biomarker to evaluate the severity of eosinophilic inflammation on the ocular surface [21]; we obtained similar results based on the clinical ocular test. In contrast, the clinical scores of patients with AKC/VKC were negatively correlated with the expression levels of *SPDEF* mRNA. These results indicate that SPDEF is another biomarker of allergic inflammation on the ocular surface and that the goblet cell density in the conjunctiva might be associated with the severity of allergic inflammation on the ocular surface.

Our study had some limitations. First, the ΔΔCT method used to analyze real-time RT-PCR data is a comparative assay and is not useful for the quantification of the results. The measurement of mRNA expression levels of ocular surface mucin in patients with allergic conjunctival diseases should thus be re-examined by quantitative PCR to determine the normal range of mRNA expression levels. Second, in the current investigation, most of the patients with AKC and VKC were males; therefore, we were not able to investigate gender differences. Since gender differences in mucin expression on the ocular surface in healthy individuals are controversial, it is necessary to investigate these using a patient group that includes a significant number of female patients with AKC and VKC. Third, the active and stable groups were classified into three clusters based on clinical scores and mRNA expression levels of *SPDEF* and *MUC16*. However, in this study, the clinical characteristics of the clusters were not further evaluated. Further studies based on a larger number of patients with allergic conjunctival diseases are necessary to clarify these characteristics. In addition, the usefulness of *SPDEF*, and *MUC16* mRNA as an ocular surface biomarker, should be validated by a population with sufficient sample size, using this study as a pilot study.

## 5. Conclusions

In summary, the levels of ocular surface mucin of patients with chronic ACDs are altered according to the stage and severity of allergic inflammation on the ocular surface. Furthermore, the clinical ocular surface test using impression cytology with filter paper might be useful to quantitatively measure the mRNA expression levels of ocular surface mucin.

## Figures and Tables

**Table 1:** Baseline characteristics of included patients.

	Stable group	Active group	*p*
No. of patients (cases)	10	9	
Age (years) (mean ± SD)	21.8 ± 12.3	29.1 ± 14.9	0.594
Sex (male : female)	8 : 2	9 : 0	0.473
Clinical score (points, median)	12	114	0.0002
Therapeutic use of immunosuppressants (yes/total)	10/10	9/9	

**Table 2:** Partial correlation coefficients between gene expression levels and allergic conjunctival disease scores.

	Clinical score	*SPDEF*	*MUC16*	Eotaxin-2
Clinical score	1			
*SPDEF*	−0.484^*∗*^	1		
*MUC16*	0.172	0.584^*∗*^	1	
Eotaxin-2	0.613^*∗∗*^	0.264	−0.198	1

SPDEF, SAM-pointed dominant-containing Ets-like factor. ^*∗*^*p* < 0.05; ^*∗∗*^*p* < 0.01.

**Table 3:** Characteristics of three cluster groups of allergic conjunctival disease patients.

	Cluster 1	Cluster 2	Cluster 3	*p* value^*∗*^
Clinical score (points) (median (range))	114 (103–223)	13 (2–23)	7.5 (3–12)	0.000003
*SPDEF* (relative expression level) (median (range))	1.9 (1.24–4.97)	7.3 (0.74–14.9)	34.6 (2.93–64.5)	0.002
*MUC16* (relative expression level) (median (range))	0.36 (0.09–0.96)	0.33 (0.26–0.45)	1.19 (1.11–2.00)	0.00008
Eotaxin-2 (relative expression level) (median (range))	106 (3.63–433)	12.8 (0.51–65.5)	56.6 (33.2–69.4)	0.134

SPDEF, SAM-pointed dominant-containing Ets-like factor. ^*∗*^Analysis of variance.

## Data Availability

The data that support the findings of this study are available from the corresponding author, S. Y., upon reasonable request.
